# Advancing frontline early pancreatic cancer detection using within-class feature extraction in FTIR spectroscopy

**DOI:** 10.1038/s41598-024-79153-0

**Published:** 2024-11-22

**Authors:** Zheng Tang, Edward Duckworth, Benjamin Mora, Bilal Al - Sarireh, Matthew Mortimer, Debdulal Roy

**Affiliations:** 1 Department of Computer Science and Mathematics, Swansea University, Swansea, SA2 8PP UK; 2 ConnectomX Ltd, Oxford, OX2 9BG UK; 3 Morriston Hospital, Heol Maes Eglwys, Morriston, SA6 6NL UK; 4 Department of Chemistry, Swansea University, Swansea, SA2 8PP UK

**Keywords:** Cancer screening, Scientific data

## Abstract

This study introduces a novel approach for the early detection of pancreatic cancer through biofluid spectroscopy, leveraging a unique machine learning pipeline comprising class-specific principal component analysis (PCA), linear discriminant analysis (LDA), and support vector machine (SVM) in both real patient and synthetic data. By conducting separate PCA on cancerous and non-cancerous samples and integrating the projections prior to LDA and SVM classification, we demonstrate significantly improved diagnostic accuracy compared to traditional methods. This methodology not only enhances predictive performance but also offers deeper insights into the influence of molecular spectra on model efficacy. Our findings, validated on real patient data, suggest a promising avenue for developing non-invasive, accurate diagnostic tools for early-stage pancreatic cancer detection.

## Introduction

In 2022, there were over 510,000 new cases of pancreatic cancer globally and over 466,000 deaths worldwide, making it one of the leading causes of cancer mortality globally^1^. Pancreatic cancer is also one of the most common causes of cancer death in the UK, with a 5-year survival rate of less than 10% and a 10-year survival rate of less than 5%. With an average of 10,452 new cases of pancreatic cancer, 2016-2018, it accounted for around 9,600 deaths each year in the UK between 2017 and 2019^2^. The 5-year survival rate increases to 20% for patients with localized disease but drops to 2% for patients with distant metastases^3^reflecting that most patients are diagnosed at a late stage when the cancer is too large or has metastasized, making it inoperable or resistant to treatment^4^.. This underscores the dire need for a rapid, flexible, cost-effective, and accurate screening method to assist clinicians in diagnosing pancreatic cancer at an early stage.

One of the main modalities for early detection of pancreatic cancer is the use of biomarkers. These are biological molecules found in body fluids or tissue samples, such as urine, oral fluids, and blood serum^5^. In particular, 19-9 carbohydrate antigen biomarkers (CA 19-9) are the most widely adopted biomarker used for the diagnosis of pancreatic cancer via blood tests^6^. On the other hand, CT scans are common for diagnosing pancreatic cancer, but they are costly and invasive and tend to manifest only when patients present symptoms, at which stage effective treatment is typically difficult. Being the most extensively studied and applied biomarkers for the diagnosis of pancreatic cancer, CA 19-9 serum levels provide respectable sensitivity and specificity of around 60 90% in symptomatic patients^7^. However, they tend to be of no significant utility in asymptomatic individuals. Furthermore, CA 19-9 levels can be raised by other non-malignant diseases in symptomatic patients^8^and only 65% of patients with resectable pancreatic cancer have elevated serum levels^9^. These characteristics contributed to the unreliability of biomarker tests for early pancreatic cancer diagnosis. Thus, researchers investigated combining biomarkers and using multimolecular biochemical analysis such as Fourier Transform Infrared (FTIR) Spectroscopy^4^.

In recent years, the analysis of biofluid FTIR spectroscopy has been closely investigated by researchers and frontier clinicians in preclinical studies and deployment^10–12^. Given the complexity of the FTIR spectral data, researchers need to utilize computational methods such as combinations of statistical and mathematical procedures to extract characteristic information from the spectra^13^. With the rapid development of machine learning techniques in recent years, the utilization of FTIR spectra became increasingly more viable in terms of both computational cost and performance, measured by accuracy, sensitivity, and specificity. Sala et al. conducted a study using serum ATR-FTIR (Attenuated total reflection-FTIR) samples to discriminate cancer against healthy samples using machine learning techniques such as random forest, partial least squares discriminant analysis (PLS-DA), and support vector machine (SVM), showing overall accuracy of 85.4%-89.7%^8^. This proof-of-concept research focuses on applying various machine learning algorithms to verify the ability of ATR-FTIR spectra to discern between cancer and non-cancer samples. Another recent study^12^ investigated the application of machine learning techniques such as PCA-SVM (Principal Component Analysis - Support Vector Machine) and PCA-LDA (Linear Discriminant Analysis) on FTIR spectra acquired from blood and urine samples from non-cancer and cancer patients. The study showed that FTIR spectra samples can be correctly classified by machine learning models with a good accuracy of 90% in some cases and verified that urine can be a solid diagnostic biofluid, although it typically produces a lower classification accuracy compared to blood samples. This study focused on investigating the viability of urine samples and the effectiveness of limiting the molecular weight region of blood samples, more specifically filtered blood samples with<10 kilodalton (kDa) region.

We propose a novel approach by performing class-specific PCA on FTIR spectra and employ a PCA-LDA-SVM pipeline to further enhance the predictive power of machine learning models. Our work improves the prospect of the application of FTIR data in front-line clinical diagnosis on pancreatic caner in two ways: increasing model predictive performance and improving model interpretability. More specifically, the main contributions of our work are as follows.

- Proposed an end-to-end analysis methodology using class-specific PCA - LDA - SVM pipeline by consolidating features from two separate PCA analysis on two different classes of samples (cancer and non-cancer), this method showed increased cross-validated prediction accuracy with both whole blood and filtered blood datasets.
- Provided empirical insight into the potential correlation between the number of groups of molecules, the number of principal components, and model performance by artificially synthesizing spectral datasets using various groups of molecular spectra.

## Experimental method

### Study design and datasets

In this study, we present a machine learning pipeline using separate PCA analysis on different classes to enhance the characteristics of the spectra. This method is applicable to most common one-dimensional spectroscopy datasets in a binary classification problem setting. Given a set of FTIR data from any cohort of patients or synthesized from other spectra, our method provides a pipeline to process data, build machine learning models, and obtain training results in different metrics to evaluate both the model performance and the dataset characteristics. Ultimately, we aim to create a model for front-line clinical predictions in the real world. We use three datasets as case studies (Table 1) to demonstrate and verify our method. These include blood samples collected by our study group with ethical approval from Morriston Hospital, Swansea, UK. IRAS ID: 252525. A complete list of patients can be found in the Supplementary Information (Table S1). All methods were performed in accordance with relevant guidelines and regulations and informed consent was obtained from all participants.

FTIR spectra were acquired using a Perkin Elmer “Spectrum Two” FTIR spectrometer in transmission mode. The resolution was 4 cm$^{-1}$, and the spectra were acquired for 5 seconds with 10 accumulations over a range of 700-4000 cm$^{-1}$. For the FTIR measurement, each fraction was diluted in a 1:24 ratio with MilliQ ultrapure water before depositing 500 $\upmu$l onto a 25 mm diameter Crystran CaF2 slide, ensuring the surface was covered to the edges and left to dry overnight for analysis (Figure S1, S2). Each biofluid sample was initially filtered through a 100 kDa filter, collecting both the filtrate (permeate) and the concentrate (retentate). The filtrate was then further filtered using 50, 30, 10, and 3 kDa filters, resulting in six subsets of plasma samples. The subsets, categorized as 0-3, 3-10, 10-30, 30-50, 50-100,>100 kDa, and whole plasma, were all analyzed for comparison (Figure S3). For the full cohort, only a 10 kDa filter was used, and both whole and<10 kDa plasma were analyzed^12^.

As a result, the datasets we used for this study (Table 1) are (i) whole blood samples from 41 non-cancer patients and 31 cancer patients, totaling 72 patients, with 3 samples taken from each patient. (ii) A subset of 61 patient samples (37 non-cancer and 24 patients, with 3 samples per patient) filtered into another dataset, we take the<10 kDa window samples to compose our second dataset (filtered blood). Each FTIR sample contains 3301 dimensions in the form of an absorption value per wavenumber^12^. (iii) Finally, we synthesized sample spectra as the third dataset to verify our method, ensuring that we have full control over the synthesized data, including its origin molecular groups, feature weights, sample size, and class distribution. This approach helps us to take steps towards understanding which factors contribute to the performance of machine learning models and how the number of molecular groups correlates to the parameters of the models and analytical tools.

From a machine learning perspective, these datasets have a small number of samples acquired from a small cohort (for the synthetic dataset, we arbitrarily decide the number of samples to emulate our real patient dataset). Therefore, we need to use the feature information from both classes efficiently to achieve good performance and robustness. The proposed class-specific method utilizes class information to augment training data, which will be detailed in the following subsection. Furthermore, the two datasets taken from real patients can exhibit great complexity, as they can be subjected to multiple external factors, such as equipment error and noise. To address these potential issues, we developed the following model training and testing pipeline to maximize model performance and robustness.

**Table 1:** Comparison of datasets and 10-fold cross-validation mean accuracy (Acc.), sensitivity(Sen.), and specificity(Spec.) results and leave-one-out-cross-validation (LOOCV) accuracy for the best performing pipelines using SVM model.

**Dataset/(PCA/Two.PCAs)**	**LDA-SVM ACC.**(%)	**LDA-SVM Sen.**(%)	**LDA-SVM Spec.**(%)	**PCA-SVM ACC.**(%)	**PCA-SVM Sen.**(%)	**PCA-SVM Spec.**(%)	**PCA-LDA SVM ACC.**(%)	**PCA-LDA SVM Sen.**(%)	**PCA-LDA SVM Spec.**(%)	**PCA-LDA SVM LOOCA Acc.**(%)	**Number of (99.9% variance)**	**Sample Size**
(i) Whole blood	81.1	67.8	90.8	76.5/73.6	60.8/58.6	87.5/84.2	74.5/83.4	61.9/80.6	83.3/85.3	75/77.3	19	216
(ii) <10kDa Window	85.2	85.6	84.2	83.4/84.4	77.2/77.2	88.3/90.1	90.6/ 91.8	86.7/ 91.1	93.9/91.7	87.9/92.3	21	183
(iii) Symthetic Data	85.5	85.0	86.0	85.5/86.5	83.0/ 85.0	88.0/88.0	85.5/85.5	86.0/85.0	85.0/86.0	86/84.5	12	200

### Machine learning pipeline

Starting with data preprocessing, all spectra undergo the same procedure where a Z-score^14^identifies any obvious outliers. The samples are then preprocessed using a background correction algorithm called asymmetric least squares smoothing^15^ (ALSS). Following this, we perform batch normalization to avoid issues like vanishing gradient. Next, we perform principal component analysis (Figure S5). Since we have many more features than the number of samples, we reduce the number of dimensions to minimize overfitting and understand the variance captured by different number of principal components (Figure S6), an example of PCA loadings from whole blood dataset is available in the Supplementary Information (Figure S7). In addition to experiments with a single PCA projection on the entire training dataset then applied to the testing set, we also perform experiments with two separate PCAs on the two different classes in the training set. This results in two principal spaces that highlight the information within each class. We then fit the training subsets separately and concatenate the two resulting sets with reduced dimensions, creating a dataset with twice the number of features based on the predetermined number of principal components in each PCA. During testing time, we fit the samples, without knowing of truth labels, onto both PC spaces and concatenate the results. The intuition behind this separate PCA technique is that there is within-class feature information in the training set. By performing two separate PCAs, we manipulate the training data to capture important class-specific features. Concatenating the PCA projections from each class aims to combine the class-specific features into a single dataset. This aims to exploit the class-specific features when they are complementary and their combination enhances class separability. This technique was shown to improve the accuracy of model prediction during cross-validation tests; more details of the experiment results will be presented in later sections. After PCA, we perform linear discriminant analysis to find a projection that maximizes the separation between classes. LDA uses the class labels of the training set and focuses on maximizing the variance between classes relative to the variance within classes (Figure S8). The application of LDA after concatenating PCA projections refines the feature space to enhance class separability. Considering that our dataset consists of a limited number of samples, we selected SVM as our classifier. SVM aims to find the optimal hyperplane that separates different classes with a maximum margin and benefits from a lower-dimensional, well-separated feature space (Figure S9). Provided that the preceding steps result in a well-separated feature space, SVM can perform very well without being particularly computationally heavy.

To ensure the validity and robustness of the results, we employ three different types of testing methods. Leave-One-Out-Cross-Validation (LOOCV), 10-fold cross-validation, and an 80-20 split hold-out test. We use Leave-one-specimen-out cross-validation, which selects one patient (three samples) as testing data and trains the model on the rest of the dataset.This method utilizes the most samples in our dataset, Given the limited size of the dataset, it produces results that are potentially closest to the scenario of training on the entirety of the dataset and predicting on one set of future patient samples. The caveat is that this method is the most computationally demanding because we rotate every patient as a testing set, resulting in a large number of iterations. In contrast, the 80-20 split holdout test is a single-pass test that trains the model on 80% of the samples and tests the model on the remaining unseen 20%. This method could suffer from between-sample variability and bias, making it is less robust. However, by bootstrapping or resampling the splits, we can obtain more robust results by increasing the number of iterations, providing a flexible testing option. This allows us to perform a large number of tests without significant computational overhead, enabling tests on synthetic datasets with a potentially large number of samples. Finally, the 10-fold CV partitions the dataset into ten subsets and rotates each subset as the testing set iteratively, then takes the mean accuracy as the final performance metric. This approach strikes a balance in performance, providing moderate computational cost and good robustness. Therefore, we have chosen 10-fold CV as our main evaluation method. Using 10-fold CV as the metric, we perform a model hyperparameter search using a grid search approach to find the best set of hyperparameters for our model. The confidence values for each classification were calculated using 95% Clopper-Pearson confidence intervals^16^, more detail can be found in the Supplementary Information (Table S3).

**Fig. 1:**
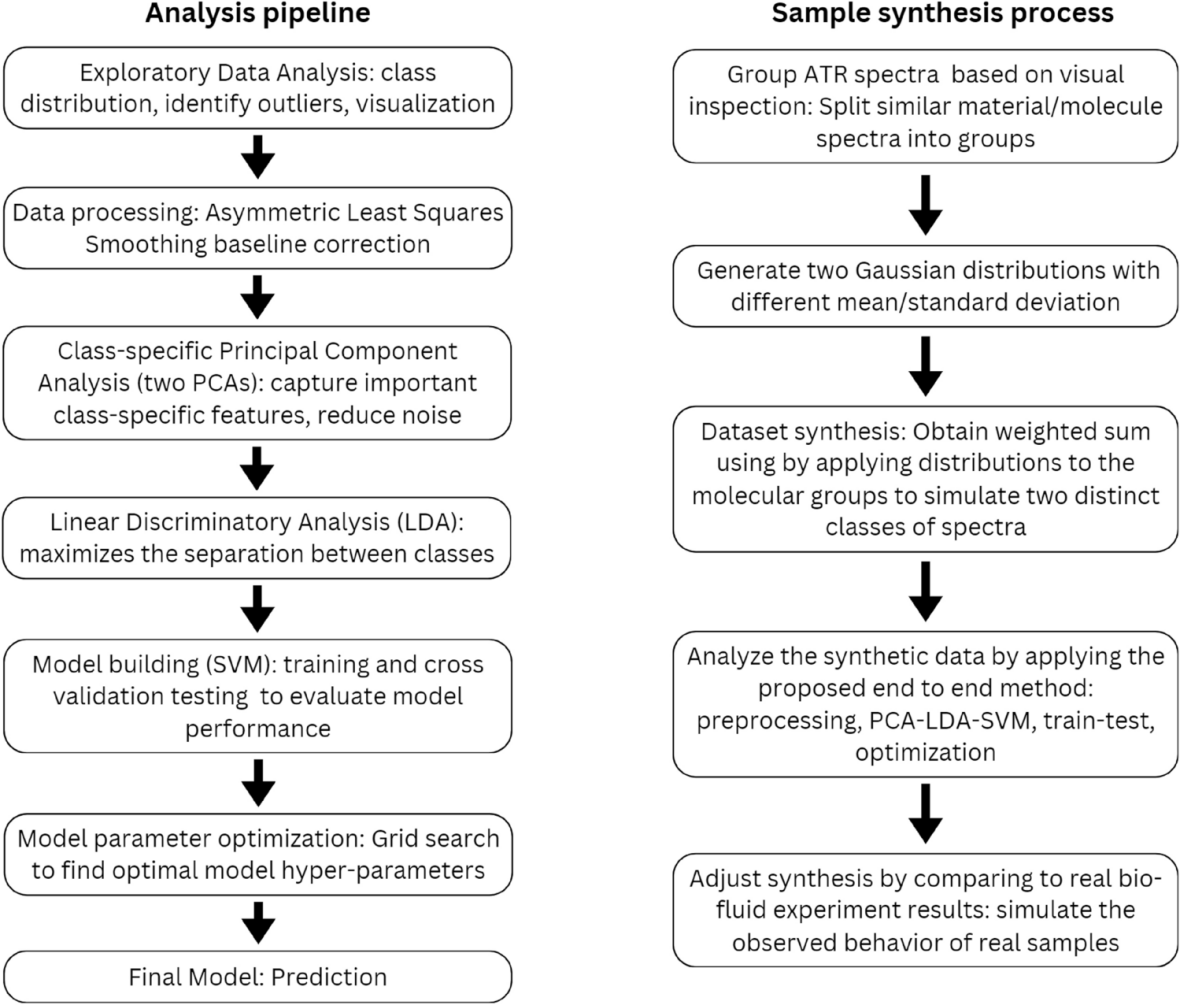
Pipelines illustrating our analysis methodology and sample synthesis process (See Table-S2 and Figures S4-S9 for technical detail).

This process can be carried out iteratively in the experiment until a desired level of performance is achieved. Figure 1 (left) shows an illustration of our proposed pipeline, further details are available in the Supplementary Information (Figure S4).

### Sample Synthesis

Due to the sensitive nature of patient data, publicly available spectroscopy datasets are scarce, especially for our specific problem. Given the limited cohort size of our datasets and the lack of knowledge of the underlying characteristics of the spectral data, we propose synthesizing spectroscopic datasets using available individual ATR spectra to assist in the analysis and help us take steps towards improved interpretability. To simulate spectroscopy with a level of complexity similar to FTIR spectra taken from patient biofluid samples, we selected 15 different groups of isolated ATR spectra samples, namely from nylon, cotton, wool, silicone, vinyl alcohol, algae, broodcomb, chitin, fur, polyester, grass, coal, silk, viscose, and amber. Each of these groups has a different number of samples, ranging from 1 to 6. Each group of spectra shares similar characteristics in terms of peaks and wavenumbers determined by visual inspection. Next, we combined the spectra in each group by averaging these spectra, each acting as a seed or component molecule spectrum. We then generate a list of 15 scalar values corresponding to the weights of each of the 15 groups. From there, we generate an arbitrary size of *N* scalar weights taken from a Gaussian distribution of our design. Combined, they represent *N* sample weights for each class (e.g., cancer and non-cancer). We then took the weighted sum of the 15 different groups of spectra from the generated weights to create the final set of synthetic spectra samples.

**Fig. 2:**
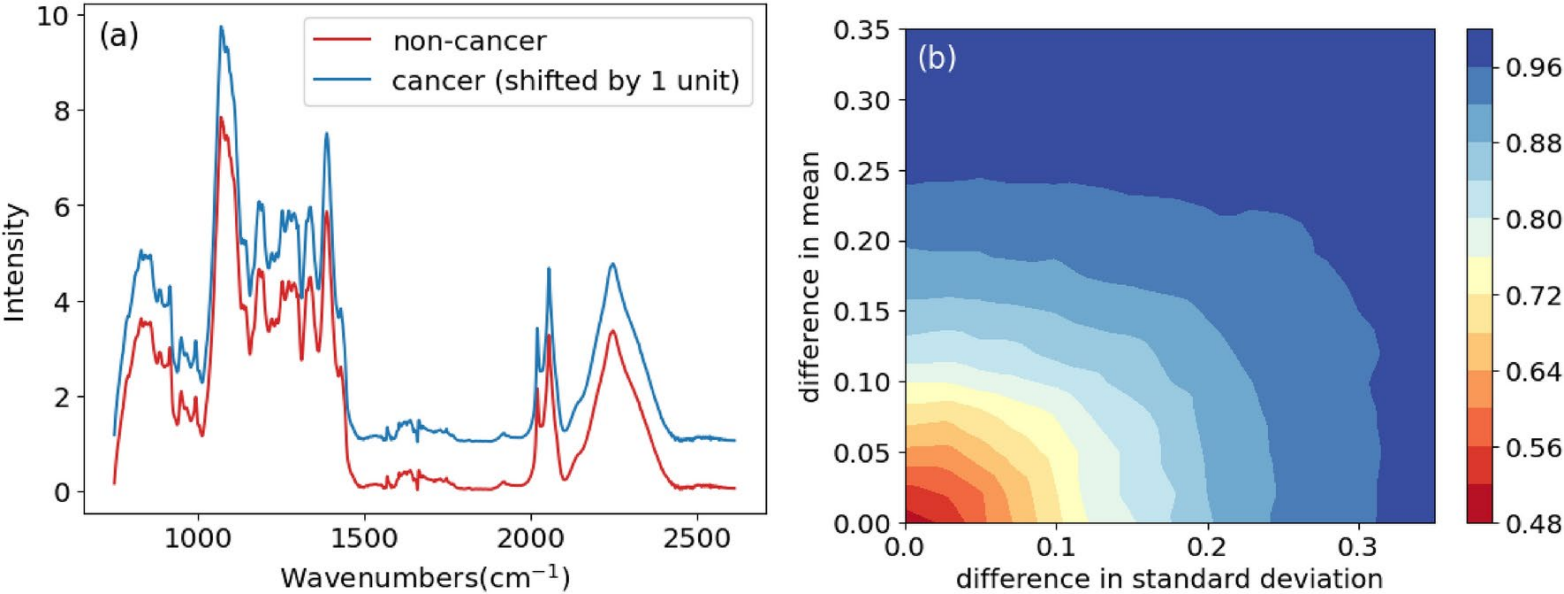
(**a**) Average spectra of the two different classes (Red: class 0 - non-cancer, Blue: class 1 - cancer + 1 unit) and (**b**) contour plot showing correlation between accuracy and differences in mean and standard deviation. Note the both spectra share similar features on the left subplot (original mean spectra). It suggests that discerning between the two by eye is highly difficult.

The discernibility between the two artificially established classes of samples depends mainly on the definition of the two Gaussian distributions, whose characteristics are determined by their mean and standard deviations. Depending on the difference in mean and standard deviation, the machine learning models demonstrate different levels of performance. As shown in Figure 2 (b), the contour plot displays the precision obtained from SVM models built using an 80/20 train-test split. This provides a general indication of the correlation between performance and the difference in distributions. Guided by the contour plot, we can select the two distributions with specific differences in mean and standard deviation to generate synthetic datasets that simulate the performance and behavior of real datasets. As an example, we synthesize a dataset with two distributions with different means and spreads, indicating that the end-spectra samples we obtain by matrix multiplication will have differently scaled features relative to one another. As shown in Figure 2 (a), it can be visually observed that the average spectra of the two different classes of synthetic samples (colored red and blue for class 0 - non-cancer and class 1 - cancer respectively) have a slight feature differences. We shifted the intensity value of class 1 by 1 unit to show a better overview of the average spectrum. In general, the 15 chosen groups of molecular spectra are assigned different weights from two normal distributions to form the final samples. Finally, we add random noise to both classes to simulate the quality of real-world data.

## Case studies and results

We define our problem as a two-class classification problem (binary classification), with the objective of distinguishing between non-cancer and cancer samples. Table 1 shows the results of the experiment from different configurations with the three datasets. By controlling the distinguishability between the two classes using the mean and standard deviation of the class distributions in the synthetic dataset, we artificially obtain a performance level that is high but close to the blood sample experiments. Specifically, we compare the 10-fold cross-validation mean accuracy obtained from three different methods: LDA-SVM, PCA-SVM, and PCA-LDA-SVM. We use the minimum number of principal components that retain 99. 9% of the variance to maintain consistency. Then when PCA is applied, we compare the results of traditional single PCA and the proposed class-specific PCA method, in this case termed “Two PCAs” which represents performing two separate PCAs on the two classes in our binary classification problem.

We observe that the class-specific PCA-LDA-SVM pipeline performed best with both whole blood and filtered blood datasets, resulting in mean accuracies of 83.4% and 91.8%, respectively. For the synthetic dataset, with a mean difference of 0.12 between the two classes, all methods achieved comparable results. For the filtered blood dataset specifically, which was shown to be the the optimal dataset in terms of performance in previous studies^12^, PCA-LDA-SVM method outperforms the other configurations by at least 5% with either one PCA or two separate PCAs. Furthermore, when we compare two PCAs with one PCA in otherwise identical configurations, two PCAs produced superior results in most cases. For the filtered blood dataset, two PCAs produce better results in every configuration, with improvements ranging from 1% to 4.4%, except for mean specificity. For the whole blood dataset, only the PCA-SVM method showed a decrease in mean accuracy when performing the Two PCAs method. Similarly, both approaches yield results comparable to those of the synthetic dataset.

**Fig. 3:**
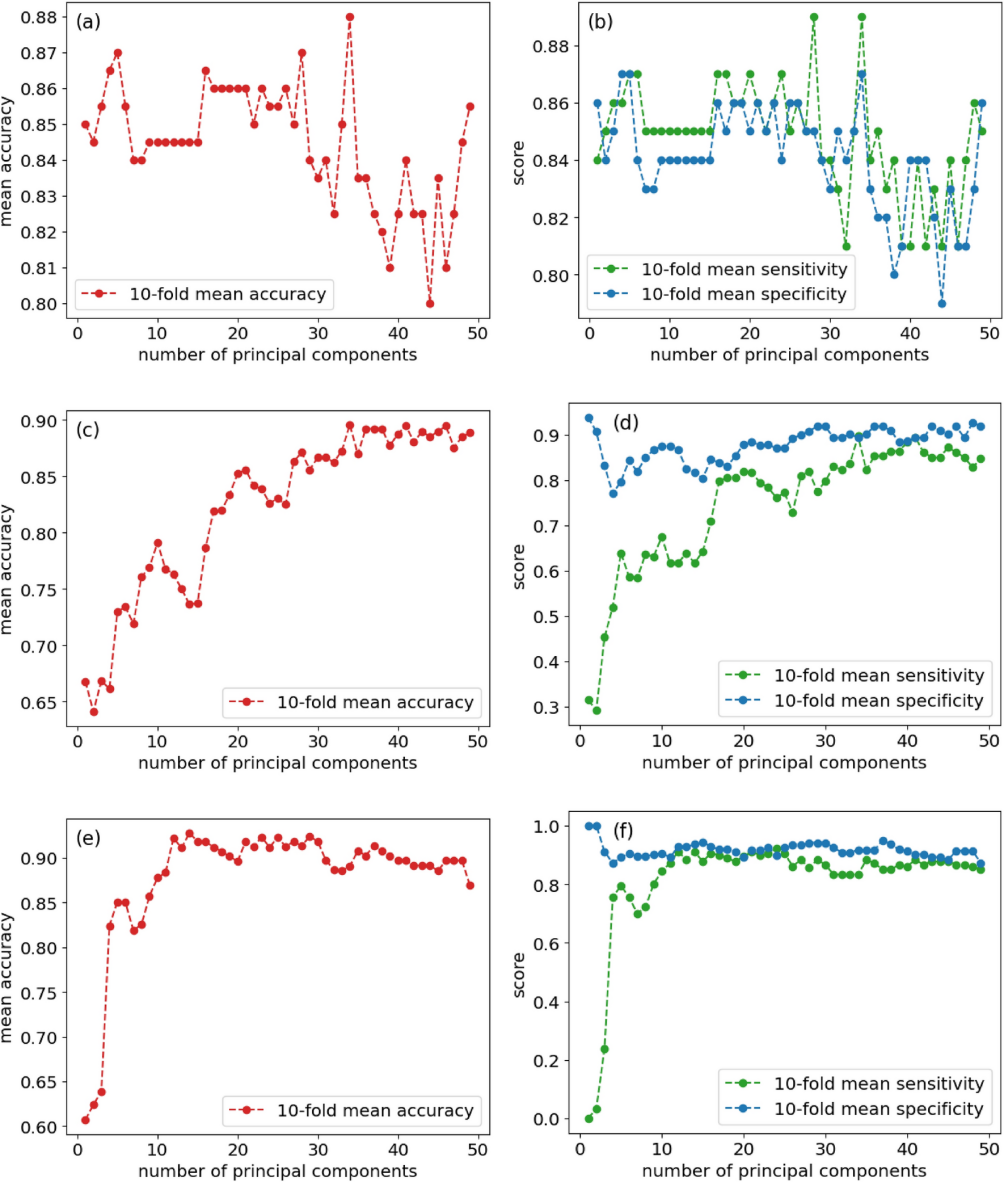
(**a**) Synthetic data accuracy vs number of PCs, (**b**) Synthetic data sensitivity/specificity vs number of PCs, (**c**) Whole blood accuracy vs number of PCs, (**d**) Whole blood sensitivity/specificity vs number of PCs, (**e**) Filtered blood performance vs number of PCs, and (**f**) Filtered blood sensitivity/specificity vs number of PCs.

As observed in the filtered blood data experiments, there is sometimes a trade-off between sensitivity (true positive rate) and specificity (true negative rate). This can also be seen in the following analysis, which explores the relationship between the number of principal components and model performance. By identifying the optimal number of principal components, we can find the respective optimal number for our three different cases, as shown in Figure 3. The number of principal components and the model’s accuracy performance share predictable patterns, with accuracy increasing as the number of PCs increases. This reflects the fact that the first few principal components encode the majority of the variance of the dataset. The accuracy then plateaus as the number of principal components increases, each within a range of optimal values. This pattern is also observed in the sensitivity and specificity score plots. Additionally, we observe a sensitivity-specificity trade-off when the number of principal components reaches a specific value. The filtered blood data converges faster than the others, achieving the best performance around 16 principal components, which could indicate that the filtered blood dataset has the least amount of noise or redundant information.

### Discussion

Empirical tests on real patient data have shown that the pipeline of applying PCA separately to two classes, concatenating projections, followed by LDA and then SVM outperforms more conventional methods from previous work, such as PCA-SVM. Specifically, the proposed method achieved a 10-fold cross-validated mean accuracy of 91.8%, with a 95% confidence interval of ±5%, compared to 83.4% for PCA-SVM and 90.6% for single PCA-LDA-SVM. In LOOCV, which offers a more representative estimate of model performance in real-world scenarios, the proposed method yielded an accuracy of 92.3%, compared to 87.9% for single PCA-LDA-SVM.

Several factors contribute to this success. By performing PCA separately for each class, the method captures the most relevant variance for each class, leading to a more effective dimensionality reduction. This tailored approach improves classification performance by providing more discriminative features that respect class boundaries. Conventional PCA, applied to the entire dataset, may dilute these features by focusing on the maximum variance across all samples, regardless of class labels. Additionally, applying PCA separately for each class might reduce noise, retaining discriminative features unique to each class. The synergy between LDA and class-specific features further enhances this method, as LDA maximizes class separability when applied to class-specific PCA projections, creating an optimal feature space for SVM to function effectively. This suggests that the preceding PCA steps create a feature space well suited for LDA to identify the most discriminative linear combinations of features. The nature of our real patient FTIR dataset involves complex class-specific patterns that are captured and utilized more effectively through this multistep approach than by a more generalized feature extraction. Furthermore, SVM’s effectiveness in this pipeline reaffirms its strength in dealing with high-dimensional data, especially when the feature space has been optimized for discrimination.

Moreover, we address the class imbalance in our cohort, which consists of a larger number of healthy patients than cancer patients, with the SVM’s “balanced” class_weight parameter, this method adjusts class weights inversely proportional to their frequencies, further enhances specificity and accuracy for the minority class. Synthetic data experiments corroborate these findings by providing insights into model performance relative to dataset composition, despite the limitations of fully replicating the intricacies of real patient data. When using synthetic data, it was observed that increasing the number of principal components could introduce noise or redundant information, negatively impacting the model performance. This underscores the importance of choosing the optimal number of components. For example, synthetic data sets based on a weighted sum of different molecular groups showed a mean accuracy range from 80% to 88%, demonstrating both the utility and limitations of such data.

In general, the class-specific PCA-LDA-SVM pipeline underscores the importance of tailored preprocessing and feature extraction methods, especially in complex datasets such as those involving real patient spectroscopy data. The success of this pipeline demonstrates that conventional approaches might not always capture the nuances of every dataset and that experimenting with innovative methods can uncover more effective ways to enhance classification performance. Empirical validation of this pipeline suggests a promising direction for future research, especially in fields where class-specific patterns play a crucial role in predictive modeling.

### Limitation

Although this study demonstrates significant advances, several areas warrant further exploration. The class-specific PCA, LDA, and SVM pipeline shows great promise but may require customization for optimal performance on diverse datasets. Although synthetic data offers valuable information, further research using real patient data will enhance understanding and model robustness. Addressing the variability inherent in the FTIR spectroscopy data, influenced by factors such as equipment calibration and environmental conditions, will further strengthen the applicability of the model. Lastly, we have a relatively small cohort of patients for this study and, although indicative of potential, the limited sample size suggests that larger and more varied datasets could provide even more reliable and generalizable results. Expanding the methodology beyond binary classification to include multiclass scenarios could greatly enhance its diagnostic utility. Future research focused on these areas could build on the strong foundation laid by this study, driving continued improvements in generalizability and broader applicability in clinical settings.

## Conclusion

The effectiveness of the class-specific PCA-LDA-SVM pipeline in diagnosing of pancreatic cancer using biofluid spectroscopy has been empirically validated, showing superior accuracy over conventional approaches used in previous studies. This study highlights the importance of novel, tailored preprocessing and feature extraction techniques for handling complex datasets, such as those encountered in medical diagnostics. The proposed method’s ability to preserve class-specific features and reduce noise has been crucial in achieving high classification performance. The integration of synthetic data alongside real patient samples in our class-specific PCA-LDA-SVM pipeline has demonstrated the potential to measure and validate performance, conduct more extensive analysis, and gain a deeper understanding of machine learning models. Importantly, we are taking deliberate steps toward unraveling the complexities of the linkage between machine learning models and real patient data, with the goal of improving model interpretability. This commitment paves the way for more transparent, explainable, and trustworthy machine learning tools in clinical applications, ensuring that advances in machine learning technology translate into tangible benefits for patient care.

## Supplementary Information

Below is the link to the electronic supplementary material.

Supplementary Information.

## Declarations

### Data Availability

The synthetic data that support the findings of this study are available on request from the corresponding author, Z. Tang. Implementation of the synthetic samples case study can be found at: https://github.com/TboneZheng/Class-specific-PCA-with-synthetic-data.git Biofluid data, including serum samples, are not publicly available due to privacy and ethical restrictions, as the data set contains information that could compromise the privacy of research participants.
