# Supplementary material for: Advancing frontline early pancreatic cancer detection using within-class feature extraction in FTIR spectroscopy
Source: Sci Rep. 2024 Nov 22;14:28940. doi: 10.1038/s41598-024-79153-0 (PMC11584613; doi:10.1038/s41598-024-79153-0)
Supplement: Supplementary file 1 — Supplementary Information. [file 41598_2024_79153_MOESM1_ESM.docx]

**Advancing Frontline Early Pancreatic Cancer Detection using Within-Class Feature Extraction in FTIR Spectroscopy**

Zheng Tang^a^, Edward Duckworth^d^, Benjamin Mora^a^, Bilal Al−Sarireh^c^, Debdulal Roy^b^ , Matthew Mortimer^c^

Zheng.Tang@swansea.ac.uk, [Deb.Roy@swansea.ac.uk](mailto:Deb.Roy@swansea.ac.uk)

a Department of Computer Science and Mathematics, Swansea University, SA2 8PP, UK
b Department of Chemistry, Swansea University, Swansea SA2 8PP, UK
c Morriston Hospital, Heol Maes Eglwys, Morriston, SA6 6NL, UK d ConnectomX Ltd, Oxford, OX2 9BG, UK

Contents

[Abstract 1](#_Toc158033386)

[1.Pancreatic Patient Information 2](#_Toc158033387)

2. Sample preparation………………………………………………………………………….5

[3. Sample synthesis 7](#_Toc158033388)

[4. Methodology 8](#_Toc158033390)

[5. PCA, LDA, and SVM 9](#_Toc158033395)

[6. Cross validation and Metrics 14](#_Toc158033396)

[7. Code and replicability 16](#_Toc158033397)

# Abstract

In this supplementary information document, we provide the following material and information to support the main manuscript with more detail to help readers get a clearer picture of our methodology and to support replicability of our results. In sections 1-3 we supply more details about our experiments including how were the patient samples prepared, the process of synthesising data from molecular ATR spectra, and we outline our proposed method including the exact pipeline the data went through which includes pre-processing, post-processing, training, and inference. In sections 4-, we provide some technical background on the machine learning tools and methods we used to give readers context on the models and how we use them to obtain prediction results and how we evaluate the performance using cross validation techniques. Finally, we provide a code repository including experiments with synthetic data and instruction on how to reproduce our results.

#

# 1.Pancreatic Patient Information

The general principle for the colour codes: green – predicted correctly with high probability, yellow – predicted correctly with medium probability and red- predicted wrong. More specifically, for Elisa the numbers are a reflection of the concentration of Sial Lewis (Ca-19.9) in the patient’s blood, higher concentrations than 59 U/ml suggesting the patient has pancreatic cancer. More specific ranges of CA19-9 for each diagnostic group (both for ELISA and FTIR) were supplied: 2-30 U/ml classifies benign (Group 3), 33-58 U/ml classifies control (Group 4), 59-81 U/ml classifies early (resectable) cancer (Group 1), and >82 U/ml classifies advanced (severe) stage (Group 2). The same classification has been used for the FTIR diagnosis column. Green indicates the correct diagnosis and patient group. Yellow indicates a sample was correctly diagnosed as cancer/non-cancer but was in the incorrect group. Red indicates incorrect diagnosis. For FTIR the number indicates the certainty of the model’s prediction. Above 0= cancer, below 0 =non-cancer. Yellow indicates that at least one of the 3 repeats was diagnosing incorrectly, but they averaged out to a correct diagnosis. PDAC: pancreatic ductal adenocarcinoma, IPMN: intraductal papillary mucinous neoplasm.

Table-S1: Table showing patient profiles (anonymous) with the initial diagnosis, final diagnosis, information from ELISA tests and information from our FTIR tests.

| Initial diagnosis | | Final Clinical Diagnosis | Age |  | Sex | ELISA number | Grouping by ELSIA | FTIR number | Grouping by FTIR |
| --- | --- | --- | --- | --- | --- | --- | --- | --- | --- |
|  | Group 1, Early Cancer | | | | | Correct diagnosis: 1, 2  1-2 | | | |
| 1.03 | | Resected PDAC | 73 |  | M | 110.4 | 2 | 11.0 | 1 |
| 1.04 | | Resected PDAC | 62 |  | M | 136.4 | 2 | 10.1 | 1 |
| 1.05 | | Resected PDAC | 61 |  | M | 30.06 | 3 | 9.8 | 1 |
| 1.08 | | Resected PDAC | 70 |  | M | 143.1 | 2 | 9.6 | 1 |
| 1.12 | | Resected PDAC | 68 |  | M | 19.7 | 3 | 7.5 | 1 |
| 1.14 | | Resected PDAC | 71 |  | M | 19.8 | 3 | 6.3 | 1 |
| 1.16 | | Resected PDAC | 56 |  | M | 88.8 | 2 | 3.4 | 1 |
| 1.17 | | Resected PDAC | 69 |  | M | 151.0 | 2 | 7.0 | 1 |
| 1.18 | | Resectable PDAC – Patient borderline fitness – not for surgery | 76 |  | M | 109.0 | 2 | 4.9 | 1 |
| 1.19 | | Resectable PDAC – Delayed due to COVID and larger PE | 65 |  | M | 129.4 | 2 | 5.4 | 1 |
| 1.20 | | Resectable PDAC – Resection delayed due to COVID | 72 |  | M | 155.0 | 2 | 13.1 | 1 |
| 1.21 | | Resectable PDAC – Resection delayed due to COVID | 75 |  | M | 46.4 | 4 | 4.8 | 1 |
| 1.22 | | Resected PDAC | 62 |  | M | Undetectable | - | 5.2 | 1 |
|  | Group 2, Late stage Cancer | | | | | Correct diagnosis: 1, 2 | | | |
| 1.01 | | Metastatic PDAC (Found intra-operatively) | 78 |  | F | 126.9 | 2 | 6.7 | 1 |
| 1.02 | | Locally Advanced PDAC (Found intra-operatively) | 59 |  | F | 68.4 | 1 | 5.3 | 1 |
| 1.06 | | Locally Advanced PDAC (Progressed pre-operatively) | 70 |  | F | 63.9 | 1 | 13.7 | 1 |
| 1.07 | | Locally Advanced PDAC (Progressed pre-operatively) | 67 |  | M | 125.1 | 2 | 4.2 | 1 |
| 1.09 | | Metastatic PDAC (Found intra-operatively) | 74 |  | M | 111.3 | 2 | 5.5 | 1 |
| 1.10 | | Locally Advanced PDAC (Found intra-operatively) | 64 |  | M | 60.3 | 1 | 7.9 | 1 |
| 1.13 | | Metastatic PDAC (Found intra-operatively) | 73 |  | F | 57.8 | 4 | 5.9 | 1 |
| 1.15 | | Metastatic PDAC (Found intra-operatively) | 73 |  | F | Undetectable | - | -0.2 | 3 |
| 2.01 | | Locally Advanced PDAC | 72 |  | F | 114.6 | 2 | 9.7 | 1 |
| 2.02 | | Locally Advanced PDAC | 75 |  | M | 198.2 | 2 | 4.9 | 1 |
| 2.03 | | Locally Advanced PDAC | 72 |  | F | 175.2 | 2 | 6.0 | 1 |
| 2.04 | | Metastatic PDAC | 61 |  | M | 129.3 | 2 | 9.7 | 1 |
| 2.05 | | Metastatic PDAC | 70 |  | M | 162.9 | 2 | 3.1 | 1 |
| 2.06 | | Locally Advanced PDAC | 76 |  | M | 55.9 | 4 | 5.8 | 1 |
| 2.07 | | Locally Advanced PDAC | 69 |  | F | pending |  | 14.8 | 1 |
| 2.08 | | Locally Advanced PDAC | 82 |  | F | pending |  | 12.9 | 1 |
| 2.09 | | Locally Advanced PDAC | 50 |  | - | pending |  | 13.5 | 1 |
|  | Group 3, Benign | | | | | Correct diagnosis: 3, 4 | | | |
| 3.01 | | Chronic Pancreatitis | 45 |  | F | 94.8 | 2 | -5.2 | 3 |
| 3.02 | | Chronic Pancreatitis | 42 |  | M | 74.9 | 1 | -3.8 | 4 |
| 3.03 | | Acute Pancreatitis | 80 |  | F | 61.2 | 1 | -8.7 | 4 |
| 3.04 | | Main Duct IPMN (Cyst) | 72 |  | F | 4.7 | 3 | -2.9 | 4 |
| 3.05 | | Branch Duct IPMN (Cyst) | 68 |  | F | 43.2 | 4 | -6.3 | 4 |
| 3.06 | | Acute Pancreatitis | 68 |  | M | 23.3 | 3 | -13.2 | 4 |
| 3.07 | | Pancreatic Cyst | 51 |  | M | 73.1 | 1 | -14.6 | 4 |
| 3.08 | | Pancreatic Cyst | 34 |  | F | 18.5 | 3 | -2.9 | 4 |
| 3.11 | | Acute Pancreatitis | 80 |  | F | 15.9 | 3 | -4.1 | 4 |
| 3.12 | | IPMN (cyst) | 48 |  | F | 20.9 | 3 | -1.3 | 4 |
| 3.13 | | Acute Pancreatitis | 64 |  | M | 43.7 | 4 | 0.1 | 2 |
| 3.14 | | Acute Pancreatitis | 67 |  | F | 8.6 | 3 | -7.7 | 4 |
| 3.15 | | Chronic Pancreatitis | 40 |  | F | Undetectable | - | -4.8 | 4 |
| 3.16 | | Chronic Pancreatitis | 52 |  | M | 17.8 | 3 | -4.5 | 4 |
| 3.17 | | Acute Pancreatitis | 70 |  | F | 40.6 | 4 | -6.6 | 4 |
| 3.18 | | Acute Pancreatitis | 69 |  | M | Undetectable | - | -5.0 | 4 |
| 3.19 | | Acute Pancreatitis | 59 |  | F | 42.9 | 4 | -6.5 | 4 |
| 3.20 | | Acute Pancreatitis | 50 |  | F | 34.8 | 3 | -11.9 | 4 |
| 3.21 | | Acute Pancreatitis | 43 |  | M | 10.4 | 3 | -10.4 | 4 |
| 3.22 | | Chronic Pancreatitis | 68 |  | M | 46.9 | 4 | -10.2 | 4 |
| 3.23 | | Acute Pancreatitis | 42 |  | M | 36.1 | 3 | -9.1 | 4 |
| 3.24 | | Chronic Pancreatitis | 60 |  | M | 10.6 | 3 | -13.8 | 4 |
| 3.25 | | Acute Pancreatitis | 44 |  | M | 43.7 | 4 | -6.6 | 4 |
| 3.26 | | Pancreatic Cyst | 69 |  | F | 19.1 | 3 | -8.5 | 4 |
| 3.27 | | Acute Pancreatitis | 55 |  | F | 15.4 | 3 | -4.4 | 4 |
| 3.28 | | Acute Pancreatitis | 86 |  | F | 15.4 | 3 | -4.1 | 4 |
| 3.29 | | Cyst IPMN | 83 |  | F | 10.6 | 3 | -9.0 | 4 |
| 3.30 | | Chronic Pancreatitis | 65 |  | F | 40.6 | 3 | -14.0 | 4 |
| 3.32 | | Branch Duct IPMN | 76 |  | F | 33.7 | 3 | -5.8 | 4 |
| 3.33 | | IPMN (Cyst) | 64 |  | F | 36.6 | 3 | -13.0 | 4 |
|  | Group 4, Control | | | | | Correct diagnosis: 3, 4 | | | |
| 1.11 | | Benign Biliary Stricture (initially presumed to be PDAC) | 74 |  | F | 25.7 | 1 | -9.3 | 4 |
| 3.09 | | CDKN2A mutation | 56 |  | F | 10.3 | 3 | -12.5 | 4 |
| 3.10 | | CDKN2A mutation | 63 |  | F | 32.4 | 3 | -1.7 | 3 |
| 4.01 | | Gallstones | 65 |  | M | 23.1 | 3 | -13.6 | 4 |
| 4.02 | | Gallstones | 60 |  | F | 55.8 | 4 | -6.5 | 4 |
| 4.03 | | Gallbladder Adenomyosis | 57 |  | F | 152.5 | 2 | -7.3 | 4 |
| 4.04 | | Anal Fistula | 47 |  | F | 16.4 | 3 | -11.5 | 4 |
| 4.05 | | Incisional Hernia | 72 |  | M | 42.5 | 4 | -8.1 | 4 |
| 4.06 | | Inguinal Hernia | 67 |  | M | 14.8 | 3 | -7.9 | 4 |
| 4.07 | | Sleeve Gastrectomy (Bariatric) | 60 |  | F | Undetectable | - | -10.4 | 4 |
| 4.08 | | Gallstones | 59 |  | F | 9.2 | 3 | -9.3 | 4 |
| 4.09 | | Umbilical Hernia | 56 |  | F | 8.4 | 3 | -12.5 | 4 |

**Filtered (<10 kDa) and Whole blood datasets.**

Two datasets are derived from the patient cohort (72 patients in total). The filtered (<10 kDa) dataset came from 61 patients (3 samples from each patient for more robust results), resulting in the total of 183 filtered blood FTIR samples. The whole blood dataset was obtained from all 72 patients (3 samples each), resulting in the total of 216 whole blood FTIR samples.

Patients with only whole blood sample are: CaPa07, CaPa203, CaPa204, CaPa205, CaPa208, CaPa209, CaPa22, PmPa09, PmPa311, PmPa312, PmPa314

# 2.Sample preparation

Centrifugal filtration into molecular weight fractions followed by the deposition of serum fractions onto CaF2 discs (Figure S1). Next steps is the drying of the serum on the disks (Figure S1). Next, spectral acquisition and clean disc background subtraction. Finally, we obtain the transmission FTIR measurement.

Figure S2 provides an illustration of the employed drying technique. The 1:24 ratio was established through experimental means to ensure an adequate volume for covering the entire disc and to maintain the serum material's residue within acceptable absorbance levels for FTIR analysis after drying. There are potential errors arising from insufficient dilution steps, which may not fully prevent uneven deposition, such as the "coffee ring effect." In the case of the HMW sample, noticeable cracking occasionally occurred due to the drying process. Despite controlled acquisition locations, it's conceivable that some important spectral shifts might have been minimized due to these erratic deposition patterns. The innermost region depicted in Figure S1, chosen for its uniformity, was determined to be the most reliable spot for controlled detection, exhibiting the lowest average error in absorption at 0.45%, compared to 0.75% and 8.1% in the middle and outer areas, respectively.


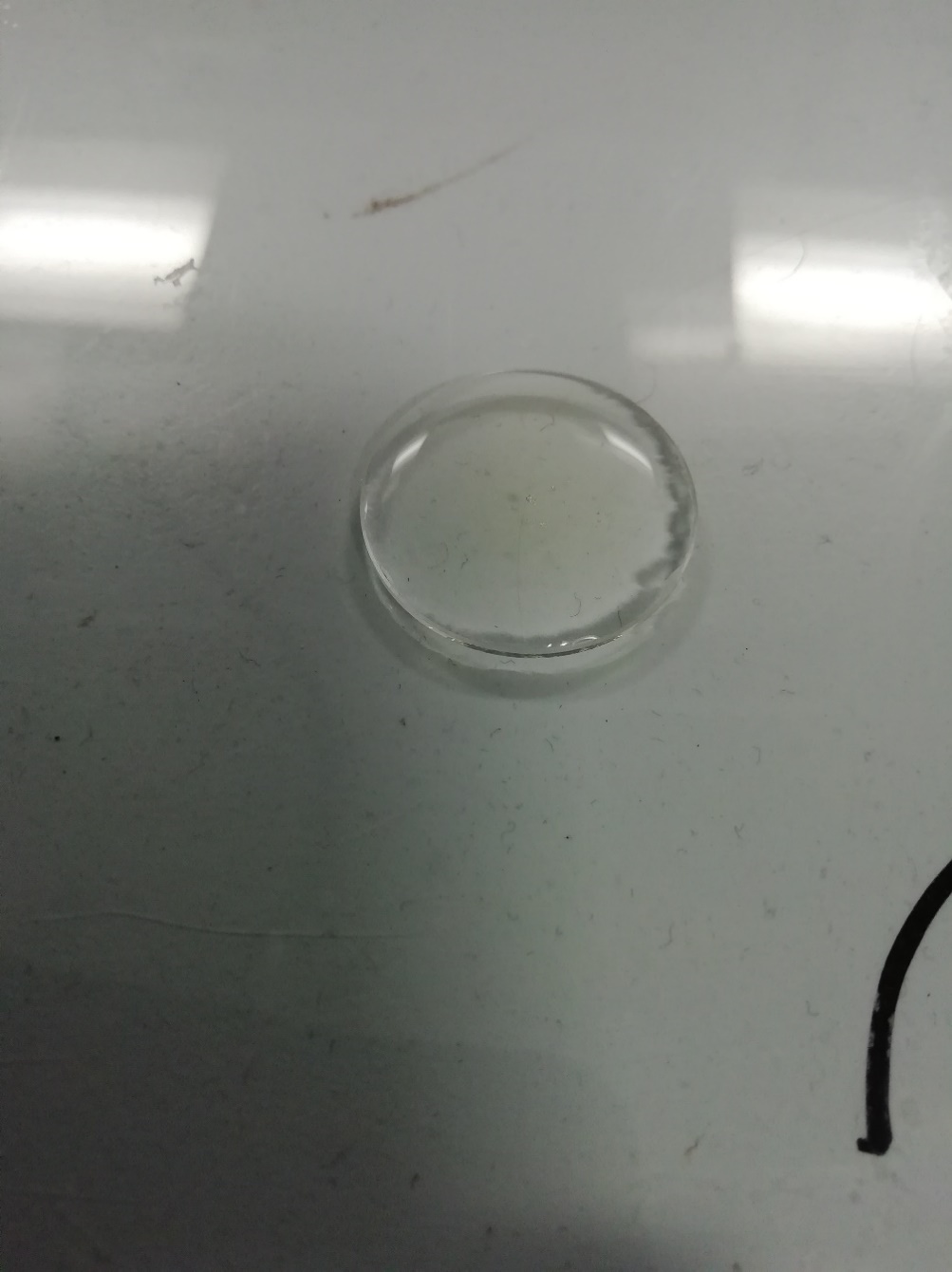

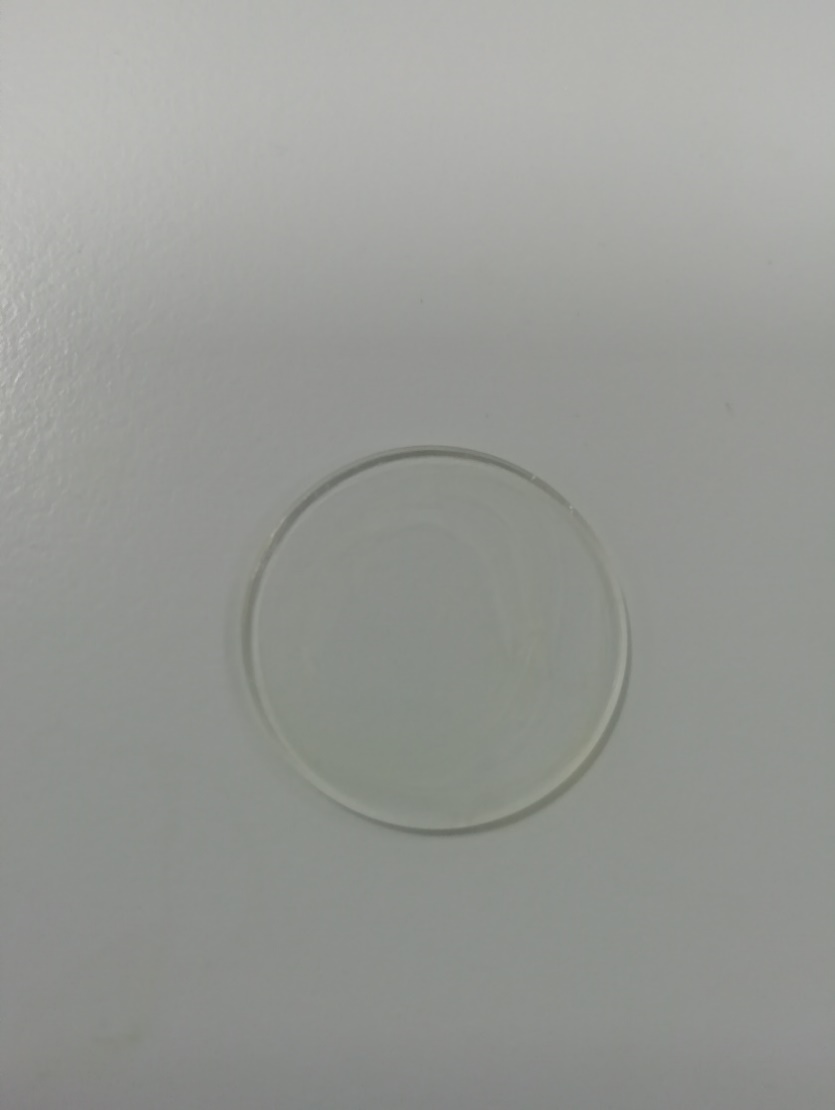

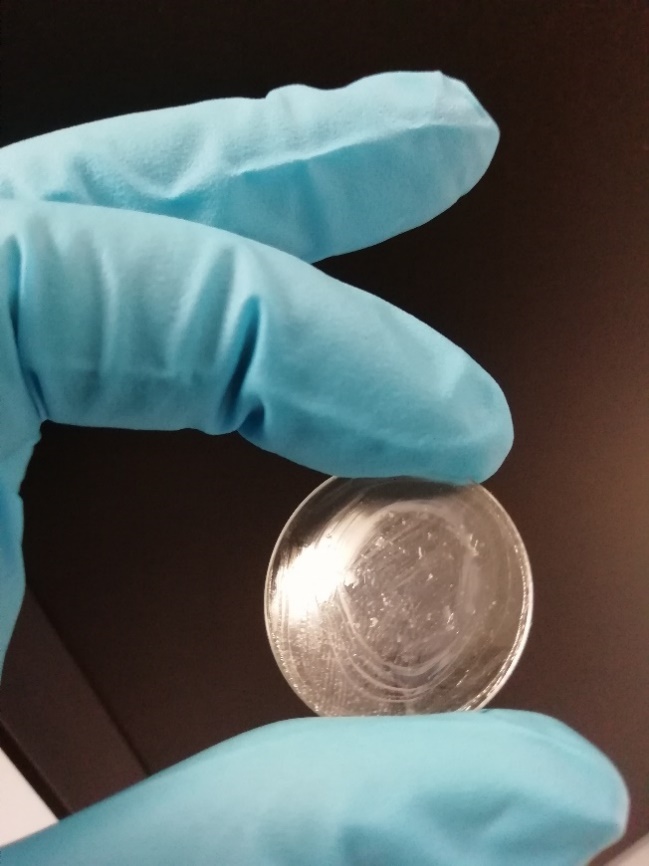


Figure-S1: images of the droplet deposition before and after drying. Serum fraction was diluted 24:1 and deposited on the disc, ensuring coverage to the edges. The layer is more even than without these precautions, but there is still evidence of the ‘coffee ring effect’ producing rings of higher weight components at the edge of the drying zone.

**FTIR instrument specifications:**

A Perkin Elmer spectrum 2 FTIR was used for the analysis and the spectral data was acquired using the integrated Perkin Elmer Spectrum software. The resolution was 4 cm^-1^, range 750-4000cm^-1^, 4 5s acquisitions. Each reading was repeated 3 times for each sample, adjusting the beam location on the disc by 2mm for each repetition. Background of a clean disc was taken beforehand for subtraction. Instrument specifications: 8,300 – 350 cm-1 long-life IR source, Dynascan™ Michelson interferometer with proprietary extended range KBr beam splitter, LiTaO3 detector and a 12mm diameter spot size. The instrument uses an over-sampling delta-sigma converte, and a small amount of zero filing and cubic spline interpolation.

#
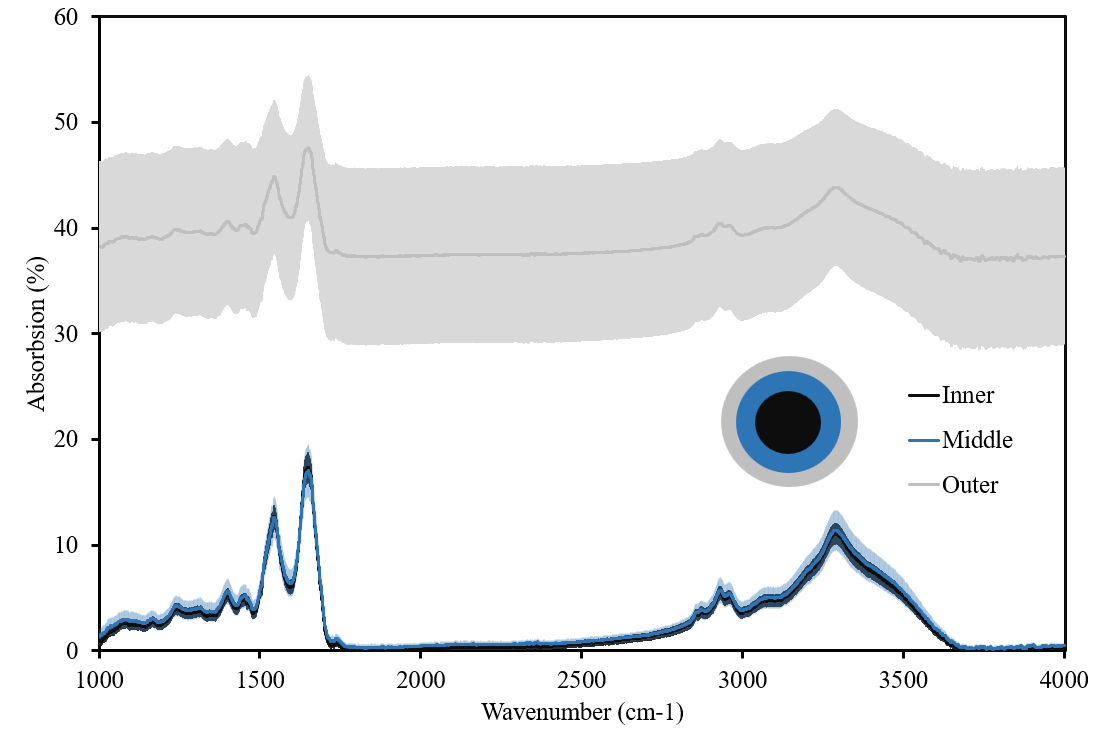


Figure-S2: raw absorption spectra averages taken from each disc section by transmission FTIR microscopy. The inner most ~8 mm, the middle ~8-16 mm and the outer ~16-25 mm diameter. One can observe the error increasing as the spectra approach the edge of the disc. The absorption is far higher at the educe due to both the coffee ring effect and contributions of the edge of the disc being within the ~3mm detection aperture.

Centrifugal Filtration for Molecular-weight Windowing

- Place samples in the top compartment of the centrifugal filtration unit
- Centrifuge for recommended time based on kDa rating (10-30 mins) at 14,000 G.
- Dilute sample into 500 µl of ultrapure water before deposition. If >100 kda concentrate use only 10 µl, up to 20 µl for the rest.
-
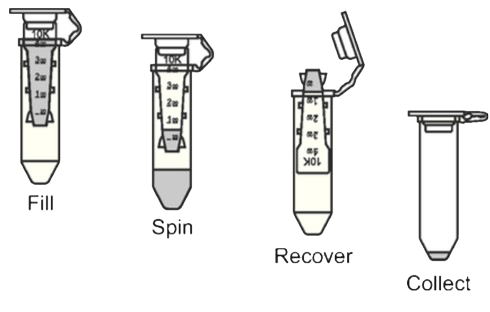
Let samples dry in ambient conditions (~2 hours) before recording IR spectra.

Figure-S3: Schematic view of concentrating samples using a Merck-Millipore centrifugal filtration unit

# 3. Sample synthesis

We selected ATR spectra samples from the following (groups) materials as listed below:

| Table S2: List of material groups and number of samples | |
| --- | --- |
| Material Group | Number of samples |
| Nylon | 5 |
| Cotton | 4 |
| Wool | 3 |
| Silicone | 2 |
| Vinyl alcohol | 3 |
| Algae | 4 |
| Broodcomb | 3 |
| Chitin | 4 |
| Fur | 2 |
| Polyester | 4 |
| Grass | 1 |
| Amber | 1 |
| Coal | 1 |
| Silk | 3 |
| Viscose | 2 |

From these groups of spectra, we take a weighted sum of each individual spectrum sample, to generate molecular group spectra. Then we generate two different distributions to simulate the difference in the two classes. We use the two parameters of a normal distribution to control the between-class discernability: mean and standard deviation. Intuitively, the mean determines the location of the data points (i.e. value) and standard deviation determines the spread of the samples. By manipulating the balance between the two parameters we can obtain a contour plot in relation to the model performance (here we simply use 80/20 split and overall accuracy):

#

# 4. Methodology

Sample preparation

Centrifugal filtration into molecular weight fractions

Deposition of serum fractions onto CaF_2_ discs

Drying of the serum on the disks

Spectral acquisition

Clean disc background subtraction.

Transmission FTIR measurement

#

Exploratory Data Analysis: calculate class distribution to understand the collected dataset, identify outliers by using Z-score, and calculate mean variance in each patient group, visualization of each class spectra to visually examine the discernability between average spectrum of each class

#

Data processing: Optional Augmentation (shift wavenumber, add Gaussian noise, linear combination), optional train-time augmentation, Asymmetric Least Squares Smoothing baseline correction to reduce the effect of background noise

#

Class-specific PCA - LDA: Reduce dimensionality and retain useful information with two PCAs on two classes. Concatenate two separate PCA projections. Perform LDA to utilize between class information.

#

Model selection (Cross Validation): Assess model general performance. LOOCV and 10-Fold CV are used to estimate general model performance (we leave each patient out in every iteration), train on the rest of the data

Model parameter optimization: iterative process to find optimal model hyper-parameters. E.g. for SVM, we optimize C: inversely proportional to the margin size and Gamma : inversely proportional to the distance

Final Model Prediction and Clinical Application

Figure-S4: Outline of the process followed for classification of patients from sample collection to final model output.

#

# **Data Pre-processing**

# **Baseline correction**: spectra were pre-processed with a background correction using the asymmetric least squares smoothing (ALSS) method. The method uses a smoothing algorithm with an asymmetric weighting of deviations to get a baseline estimator. This allows a corrective baseline to be quickly obtained while retaining the signal peak information.

**Normalization**: Baselining was followed by average normalisation by dividing by the average intensity for each spectrum.

# 5. PCA, LDA, and SVM

**Principal Component Analysis (PCA)** is a widely used statistical technique that aims to simplify high-dimensional datasets by transforming them into a lower-dimensional space while retaining the most crucial information. PCA accomplishes this by identifying the primary axes, or principal components, along which the data varies the most. These components are orthogonal to each other and ordered based on the amount of variance they capture, with the first component explaining the most variance. By projecting the data onto these components, PCA allows for dimensionality reduction while preserving the essential patterns and structures within the dataset. It finds applications in data compression, feature extraction, visualization, and noise reduction, aiding in the exploration and understanding of complex datasets in various domains. Here we use the PCA technique to reduce dimensionality and at the same time identify useful information, and furthermore, rule out information that could negatively impact model performance.


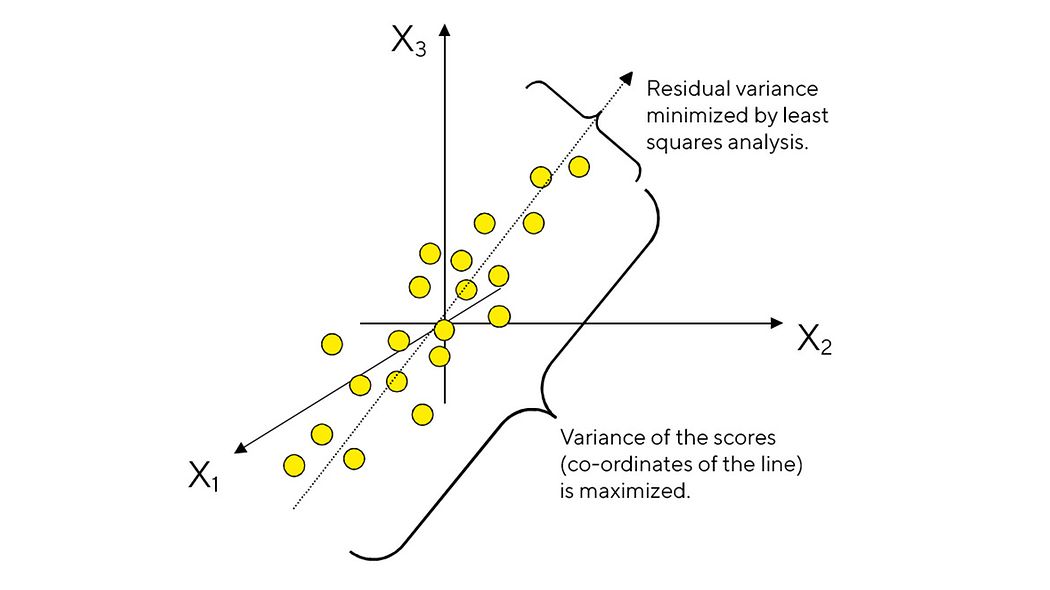


Figure S5: Schematic of principal component analysis.

**PCA analysis** is performed on datasets to examine the percentage of variance retained in the reduced dimensionality in relation to the number of principal components as shown in the figures below is the num_pc to variance plot for Whole blood dataset and the loadings of PC1, PC2, and PC3:


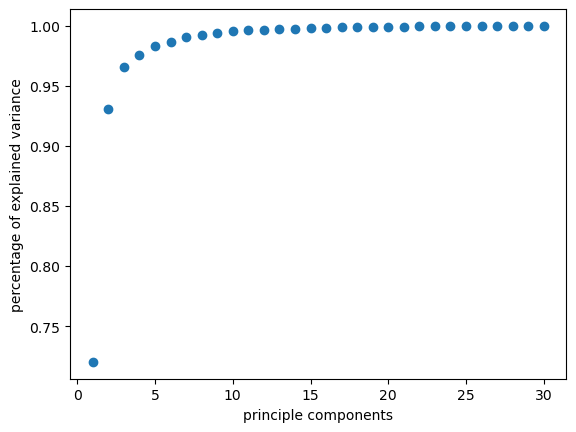


Figure S6: Plot of percentage of explained variance with the number of principal components.


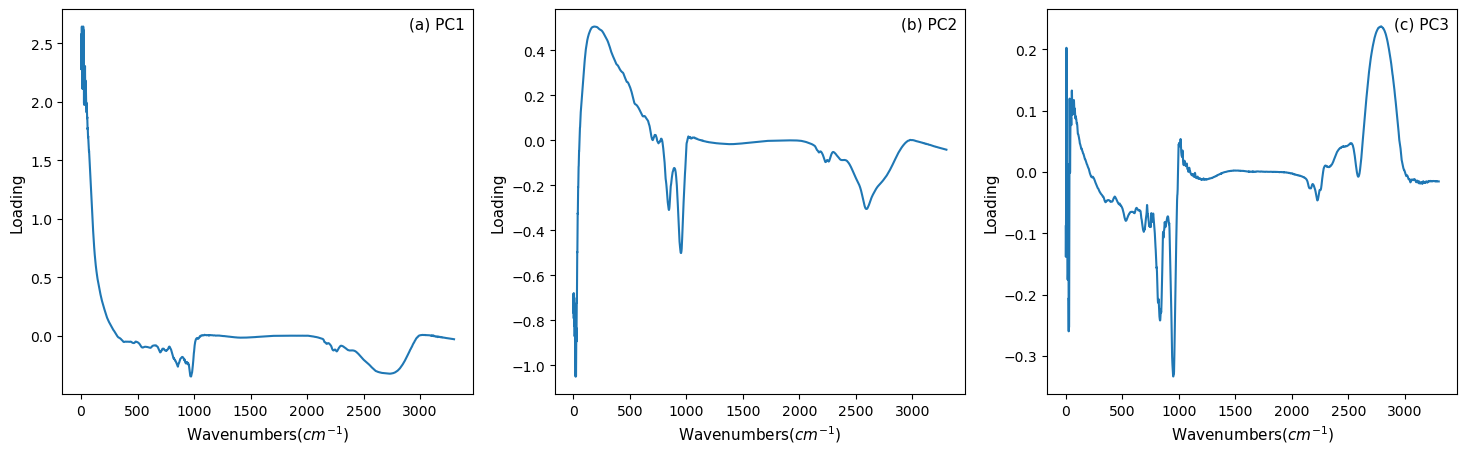


Fig S7: loadings of principal components : a) PC1, b) PC2 and c) PC3

**Specimen wise splitting before PCA** is ensure so that no data leakage ever occurs in our experiments. PCA is developed on the training split then applied to the testing data. First, we split the data specimen-wise, so that all three FTIR samples taken from each single patient is either in the training set or the testing set and never in both. Then we fit the PCA on the training dataset and use the fitted PCA to transform the testing set. For model building, we retain 99.9% variance of the original data. For Whole blood, Filtered blood, and synthetic dataset (200 samples from 15 molecular groups), 19, 21, and 12 PCs retain 99.9% respectively.

**Class-specific PCA**

Our proposed class-specific PCA method is a custom approach to Principal Component Analysis (PCA) that applies PCA individually to different classes within a dataset. This method is designed to handle cases where the data distribution varies significantly between classes, which can be a limitation when using traditional PCA.

We perform PCA on the training and testing data, but instead of applying PCA on the entire dataset at once, it does so separately for each class. For each class: First the subset of the training data corresponding to the current class is extracted. Then PCA is fitted only using the training data, retaining the specified number of principal components. Next, the class-specific PCA model is used to transform both the training and test data. Finally, the reduced training data from all classes are concatenated to form a single dataset.

**Differences from Traditional PCA**

Where in traditional PCA applies one PCA model to the entire dataset, treating all data points as coming from a single distribution. This homogeneous transformation assumes the same principal components are relevant for all data points, regardless of their class which can lead to suboptimal principal components if the data distribution varies significantly between classes, as the PCA components are influenced by the global variance structure. Whereas our class-specific PCA method applies separate PCA models to data from each class which provides a more accurate representation of the variance structure within each class, leading to potentially better performance for classification tasks involving heterogeneous data distributions.

Overall, the class-specific method offers a more nuanced and potentially more powerful approach to dimensionality reduction when dealing with multi-class datasets that exhibit significant class-specific variability.

**Linear Discriminant Analysis (LDA)** is a statistical method used in machine learning and pattern recognition for dimensionality reduction and classification. Its primary goal is to find a linear combination of features that separates two or more classes of objects or events. The methodology behind LDA involves projecting the data onto a lower-dimensional space in such a way that maximizes the distance between the means of the classes while minimizing the variance within each class. This process enhances the class separability, making it easier to classify new instances.

LDA is based on the concept of searching for linear axes that best discriminate between the classes. It assumes that the data distributions are Gaussian and that each class has the same covariance matrix. Despite these assumptions, LDA often performs well in practice even when the assumptions are somewhat violated.

The steps involved in LDA include computing the within-class scatter matrix, the between-class scatter matrix, and then finding the linear combinations of features that maximize the ratio of the determinant of the between-class scatter matrix to the determinant of the within-class scatter matrix. These linear combinations are used to transform the original features into a lower-dimensional space.

**
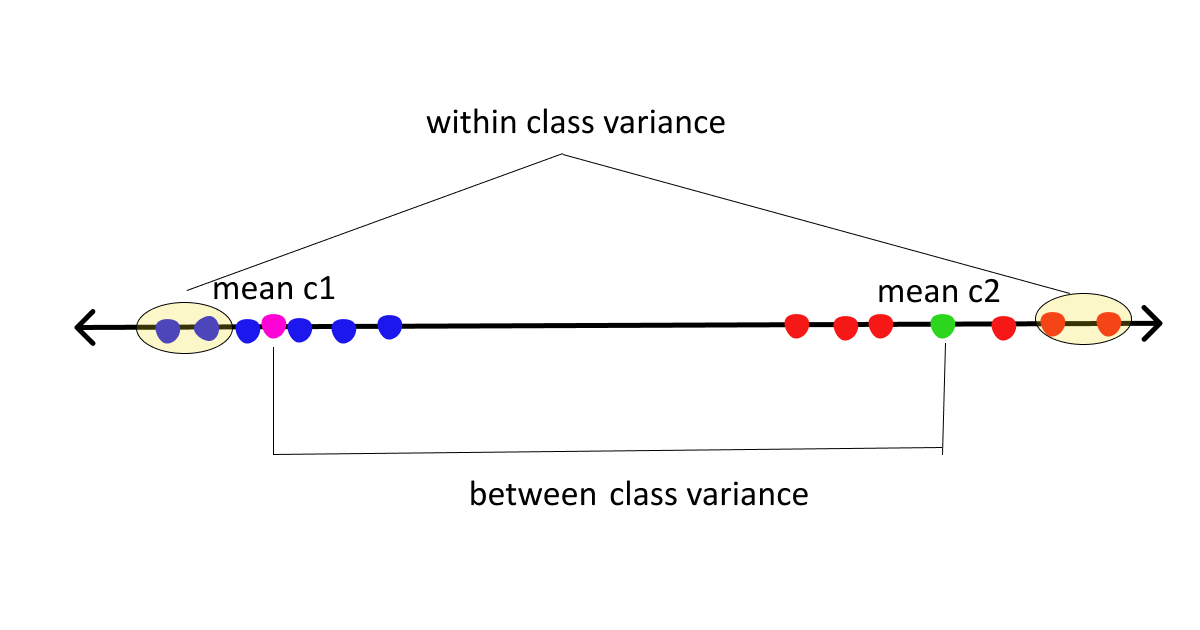
**

Figure S8: Schematic of classification by linear discriminant analysis (LDA)

**Support Vector Machine (SVM)** is a powerful supervised machine learning algorithm widely used for classification and regression tasks. It works by finding the optimal hyperplane in a high-dimensional space that best separates the different classes or captures the decision boundaries for regression. SVM aims to maximize the margin, the distance between the hyperplane and the closest data points (support vectors), providing robustness to new data. It employs a kernel trick to map the input data into a higher-dimensional space, allowing for the separation of non-linearly separable classes. SVM seeks to minimize classification errors while maximizing the generalization capability, making it effective in various domains like image recognition, text classification, and biological sciences where it demonstrates high accuracy and versatility in handling both linear and non-linear data distributions.


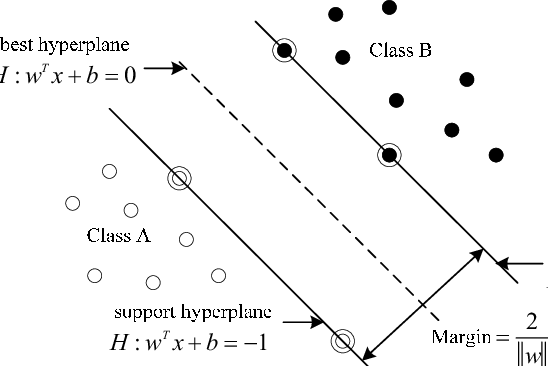


Figure S9: Schematic of classification by support vector machine (SVM)

**Class Imbalance**

# **SVM class weight:** To address the class imbalance in our datasets. We use parameter, class_weight="balanced" parameter in SVM which addresses imbalanced datasets by adjusting the weights of classes inversely proportional to their frequencies. This ensures that minority classes receive higher weights and majority classes receive lower weights, balancing the influence each class has during training. By doing so, the SVM becomes more sensitive to the minority class, improving metrics such as precision, recall, and F1-score for that class. This parameter automates the process of balancing class weights, making it easy to apply without manual tuning and helping the classifier generalize better to unseen data, particularly in imbalanced scenarios. This approach is highly effective for practitioners dealing with datasets where class distributions are unequal, ensuring more reliable and robust predictions.

# 6. Cross validation and Evaluation Metrics

**K-Fold Cross-Validation (CV)** is a resampling technique used to evaluate the performance and robustness of machine learning models by partitioning the dataset into k equally sized subsets, or folds. In each iteration, one fold is retained as the validation set while the remaining k-1 folds are used for training the model. This process is repeated k times, with each fold serving as the validation set exactly once. The model's performance is then determined by averaging the evaluation metrics (such as accuracy, error, etc.) obtained from each iteration. K-Fold CV provides a more reliable estimate of the model's performance compared to a single train-test split, offering a balance between using most of the data for training and ensuring separate validation, thereby reducing overfitting and providing a more robust assessment of the model's generalization ability. Adjusting the value of k allows for variations in the trade-off between computational complexity and accuracy estimation, making it a widely used technique in model evaluation and hyperparameter tuning.

Here we use 10-fold validation, specifically stratified group 10-fold, so that the dataset is split **specimen wise** and all three samples from each specimen is either in the training set or the testing set. This approach produces reasonably sized train and test set for each experiment and we use the mean accuracy (sensitivity and specificity) as the metrics to estimate model performance.

**LOOCV (per-specimen)** Leave-One-Out Cross-Validation (LOOCV) is another resampling technique used to assess the accuracy and robustness of machine learning models by using each individual **specimen** (all 3 samples from each patient) as a single validation set while the remaining specimens form the training set by using leave one group out cross-validation where the dataset is grouped by patient. (Note in the case of Synthetic samples, each sample is independent to one another as they are not derived from patients therefore did not need to be grouped and split per specimen like real patient samples.) This method ensures that every specimen is used once as a validation set, offering a thorough and exhaustive evaluation of the model's performance. LOOCV is particularly useful when the dataset is small, as it maximizes the training data used in each iteration. However, it can be computationally expensive and may lead to higher variance in the performance estimates. Despite these drawbacks, LOOCV provides an almost unbiased estimate of the model's generalization ability and is often used in scenarios where a highly detailed assessment of model performance is required. In our context, we use LOOCV per-specimen to ensure that the model is validated on each individual specimen, allowing us to evaluate the model's performance in a highly granular manner.

**Clopper-Pearson Confidence Interval**

Confidence intervals are an effective way to evaluate the reliability of a classification method's accuracy. A 95% confidence level is generally recommended for most purposes, although in some cases, lower or higher confidence levels might be more appropriate, especially to prevent significant negative outcomes. Table S5 includes additional results in 95% confidence interval calculation.

The Clopper-Pearson interval, also known as the exact interval, is a technique designed to address inconsistencies at extreme predictive values. This method uses the inverse of the cumulative beta probability density function to generate a confidence value similar to the Wald interval but with greater consistency at the extremes. The resulting interval typically has different upper and lower bounds. For instance, a classifier that is 100% accurate in identifying 50 cancer patients and 50 healthy patients would have a 95% confidence interval ranging from 96.4% to 100%.

Table-S3: Additional information including 95% confidence interval(%) for different data sets.


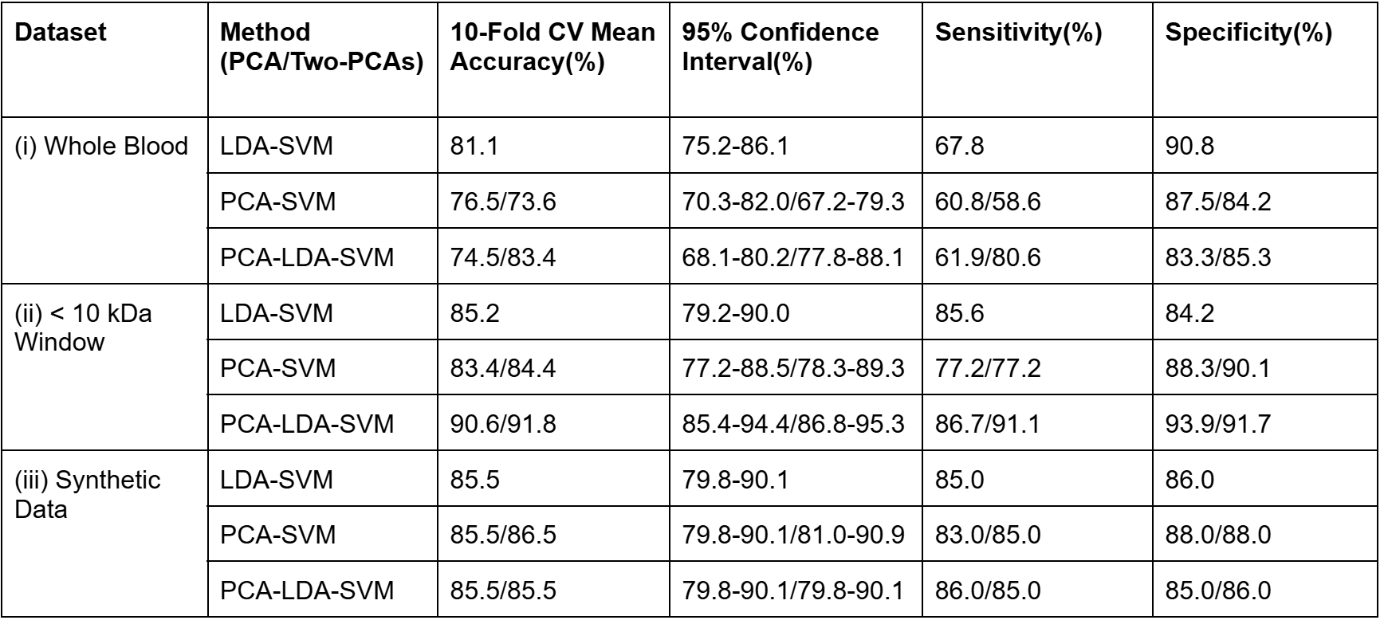


Additional information about the results in table 1, that couldn’t be fit into the main table. Accuracies are all post cross-validation.

# 7. Code and replicability

The synthetic data that support the findings of this study are available on request from the corresponding author, Z. Tang.
Biofluid data, including serum samples, are not publicly available due to privacy and ethical restrictions, as the data set contains
information that could compromise the privacy of research participants.

Implementation of synthetic dataset and proposed machine learning pipeline can be found at this repository:

https://github.com/TboneZheng/Class-specific-PCA-with-synthetic-data
